# Future scientific paradigms in the integration of materials, aerospace and information

**DOI:** 10.1093/nsr/nwaf122

**Published:** 2025-04-10

**Authors:** Cheng Wang, Yu Zhao, Shicheng Hu, He Jia, Wei Yan, Dongyu Fan, Yanhua Cheng, Wenlong Bao, Zhen Wang, Lichao Yuan, Feng Yan, Meifang Zhu, Changjun Jiang

**Affiliations:** Key Laboratory of Embedded System and Service Computing, Ministry of Education, Tongji University, China; School of Computer Science and Technology, Tongji University, China; Shanghai Artificial Intelligence Laboratory, China; Key Laboratory of Embedded System and Service Computing, Ministry of Education, Tongji University, China; School of Computer Science and Technology, Tongji University, China; Shanghai Artificial Intelligence Laboratory, China; Key Laboratory of Embedded System and Service Computing, Ministry of Education, Tongji University, China; School of Computer Science and Technology, Tongji University, China; Shanghai Artificial Intelligence Laboratory, China; Beijing Institute of Space Mechanics and Electricity, China; Laboratory of Aerospace Entry, Descent and Landing Technology, China Aerospace Science and Technology Corporation, China; State Key Laboratory for Modification of Chemical Fibers and Polymer Materials, College of Materials Science and Engineering, Donghua University, China; Beijing Institute of Space Mechanics and Electricity, China; Laboratory of Aerospace Entry, Descent and Landing Technology, China Aerospace Science and Technology Corporation, China; State Key Laboratory for Modification of Chemical Fibers and Polymer Materials, College of Materials Science and Engineering, Donghua University, China; Beijing Institute of Space Mechanics and Electricity, China; Laboratory of Aerospace Entry, Descent and Landing Technology, China Aerospace Science and Technology Corporation, China; Beijing Institute of Space Mechanics and Electricity, China; Laboratory of Aerospace Entry, Descent and Landing Technology, China Aerospace Science and Technology Corporation, China; Beijing Institute of Space Mechanics and Electricity, China; Laboratory of Aerospace Entry, Descent and Landing Technology, China Aerospace Science and Technology Corporation, China; State Key Laboratory for Modification of Chemical Fibers and Polymer Materials, College of Materials Science and Engineering, Donghua University, China; Beijing Institute of Space Mechanics and Electricity, China; State Key Laboratory for Modification of Chemical Fibers and Polymer Materials, College of Materials Science and Engineering, Donghua University, China; Key Laboratory of Embedded System and Service Computing, Ministry of Education, Tongji University, China; School of Computer Science and Technology, Tongji University, China; Shanghai Artificial Intelligence Laboratory, China

## Abstract

This perspective dissects the fourth paradigm in aerospace systems and explores how future paradigms drive collaborative advancement by integrating materials, aerospace, and information to address deep space exploration and interdisciplinary research challenges.

Scientific paradigms provide structured frameworks of theories, methods and standards that delineate legitimate contributions within a field (Fig. 1a). Their evolution has profoundly influenced how we comprehend, navigate and resolve the increasingly complex challenges of multidisciplinary collaboration. The first paradigm, experimental science, emphasized systematic observation and experimentation, as exemplified by Kepler’s laws of planetary motion. The second paradigm, theoretical science, introduced mathematical frameworks to explain natural phenomena, as represented by Newton’s law of universal gravitation. These two paradigms established the foundation of scientific inquiry. The advent of the third paradigm, computational science, transformed research by leveraging computational power to model, simulate and analyze complex systems with unprecedented precision and scale, as demonstrated by spacecraft trajectory modeling [1]. The fourth paradigm, data-intensive science, marked a transformative leap by leveraging vast datasets to uncover patterns and insights, enabling breakthroughs such as real-time satellite image processing [2]. In this perspective, we dissect the fourth paradigm for application in aerospace systems and explore how future paradigms drive collaborative advancement by integrating materials, aerospace and information.

**Figure 1:**
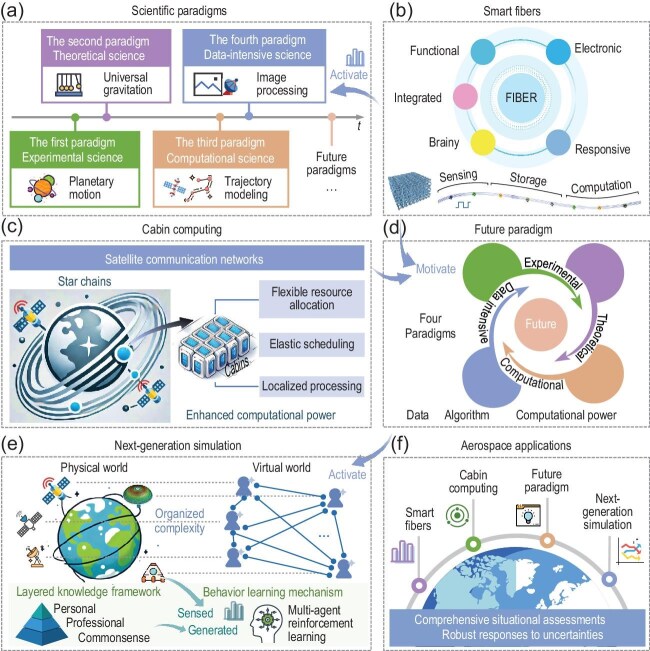
(a) Scientific paradigms. (b) Smart fibers. (c) Cabin computing. (d) Future paradigm. (e) Next-generation simulation. (f) Aerospace applications.

The fourth paradigm leverages artificial intelligence technologies, including machine learning, reinforcement learning and data fusion, to analyze large-scale data and extract knowledge and patterns [3]. Such capabilities are critical to real-time monitoring, predictive analysis and autonomous decision-making in aerospace systems [4]. However, implementing this paradigm poses a critical challenge: balancing the need for large-scale data acquisition with stringent engineering constraints on weight and energy efficiency. Traditional rigid sensing components collect data from discrete sensors or sensor arrays. Yet, the stringent lightweight requirements of a spacecraft create cascading trade-offs, as each additional sensor increases mass, energy consumption and communication complexities [5]. Moreover, traditional rigid sensing components suffer from wiring complexity, limited scalability and vulnerability to physical damage, further limiting their feasibility in large-scale and real-time aerospace networks [6]. This inherent conflict prevents traditional rigid sensing components from fulfilling the dual demands of data acquisition and system performance, constraining the broader application of the fourth paradigm in aerospace systems.

To address these challenges, innovative sensing components are required to reconcile extensive data acquisition with the stringent constraints of aerospace engineering. Recently, Broer *et al.* [7] highlighted the significance of multi-sensor fusion in the structural health monitoring of composite aircraft. Bombiński *et al.* [8] proposed a tool condition monitoring system for the aerospace industry, integrating sensing and data processing to meet high-precision and reliability requirements under strict constraints. Building upon these advancements, smart fibers offer a promising solution [9]. By combining advanced material properties with integrated computational capabilities, smart fibers enable comprehensive sensing while maintaining lightweight characteristics crucial for aerospace applications. The FIBER (functional, integrated, brainy, electronic and responsive) concept proposed by Professor Meifang Zhu of Donghua University provides a theoretical foundation for designing smart fibers tailored to aerospace needs (Fig. 1b) [10]. With multifunctional sensing, storage and computation capabilities, smart fibers present significant advantages for achieving ‘high coverage’ and ‘low weight’. Incorporating microscale chips allows fibers to form computational clusters, supporting localized data processing. Smart fibers can address traditional storage limitations by utilizing computing-in-memory technology, enabling real-time, on-site data processing without extensive pre-processing. By integrating data acquisition, processing and storage, smart fibers activate applications of the fourth paradigm in aerospace systems, enhancing real-time sensing, risk prediction and system optimization.

Although smart fibers activate the data-intensive science of the fourth paradigm, their hardware architecture limits storage and computational capacity. A robust computing model is required to address these challenges associated with large-scale data processing and dynamic mission requirements. In this perspective, Professor Changjun Jiang from Tongji University proposed cabin computing as a solution [11]. Cabin computing leverages the strengths of grid computing and cloud computing. By integrating high-performance distributed processing from grid computing with the on-demand scalability of cloud computing [12], cabin computing offers a cross-domain and mission-centric environment for computational integration, supporting flexible deployment and efficient resource allocation (Fig. 1c). Utilizing mobile special cabins on star chains interconnected via satellite communication networks establishes a highly efficient and scalable sky computing network. This model facilitates the flexible configuration of cross-domain resources for collaborative operations. Furthermore, with autonomous operation and maintenance capabilities, cabin computing adapts dynamically to mission requirements by supporting flexible deployment, resource scaling and automatic decommissioning of computational environments upon task completion. With its flexible resource allocation, elastic scheduling and localized processing, cabin computing enhances computational power for aerospace systems while ensuring the security of sensed data throughout transmission, storage and utilization.

Smart fibers enable real-time data acquisition, and cabin computing

provides the computational foundation exemplifying the fourth paradigm, essential for advancing aerospace applications. Although existing paradigms perform well in controlled environments, they struggle to address rapidly changing missions and unforeseen challenges of complex environments such as deep space. Specifically, technical bottlenecks in real-time processing and risk prediction hinder the resolution of organized complexity issues, motivating further paradigm evolution [13]. Therefore, a new integrated paradigm—the future paradigm—is required, characterized by the deep integration of data, algorithms and computational power across four existing paradigms, incorporating experimental, theoretical, computational and data-intensive capabilities to enable cross-disciplinary synergy (Fig. 1d). This comprehensive paradigm is crucial for addressing the unique challenge of aerospace systems, enabling transformative advancements in deep space exploration and complex mission environments.

Within the future paradigm, we propose next-generation simulation technology to achieve unprecedented capabilities in aerospace systems (Fig. 1e). By bridging physical and virtual worlds, the future paradigm activates next-generation simulation to model and analyze systems exhibiting organized complexity. This technology comprises two components: the layered knowledge framework and the behavior learning mechanism, which power dynamic, interactive and adaptive simulations. The layered knowledge framework hierarchically integrates three levels of knowledge: commonsense, professional and personal. Commonsense knowledge provides general reasoning capabilities, while professional knowledge contributes domain-specific expertise from fields such as materials, aerospace and information. Personal knowledge adds individualized adaptability for agents. This framework enriches raw sensed data from smart fibers with generated data, bridging sensor-induced data gaps and enabling nuanced behavior modeling for comprehensive system understanding. Then, the behavior learning mechanism, based on multi-agent reinforcement learning, allows agents to interact dynamically with each other and environments [14]. Leveraging enriched data from the layered knowledge framework, agents learn optimal strategies through iterative feedback, continuously enhancing decision-making and predictive abilities. These components enable simulations to reveal hidden risks, predict system behavior and adapt to unforeseen challenges, driving advancements within the future paradigm. In aerospace systems, next-generation simulation technology offers transformative benefits, including enhanced situational awareness, improved risk prediction and optimized operational efficiency.

Smart fibers provide real-time sensing data that activate the fourth paradigm. Cabin computing delivers enhanced computational power, and the future paradigm, which integrates four paradigms, activates next-generation simulation technology. This synergy facilitates seamless data flow, robust computational support and actionable insights for complex mission environments, enabling comprehensive situational assessments of aerospace systems and robust responses to uncertainties (Fig. 1f). Ultimately, the integration of materials, aerospace and information fosters future scientific paradigms. This convergence paves the way for innovative solutions to the intricate challenges of deep space exploration and interdisciplinary research.

## FUNDING

This work was supported by the National Natural Science Foundation of China (62372328), the Fundamental Research Funds for the Central Universities (22120240357 and 2024-1-ZD-04), the Program of Shanghai Academic Research Leader (22XD1423700), the National Key Research and Development Program of China (2022YFB4501704), and the Leadership Project under the Oriental Talent Program.
